# A green hydrophobic deep eutectic solvent for extraction of phenol from aqueous phase

**DOI:** 10.1038/s41598-023-44600-x

**Published:** 2023-10-14

**Authors:** Javad Saien, Mansoureh Bahiraei, Farnaz Jafari

**Affiliations:** Department of Chemistry and Petroleum Science, Bu-Ali Sina University, Hamedan, Iran

**Keywords:** Pollution remediation, Chemical engineering

## Abstract

Deep eutectic solvents (DESs), have been recognized as effective materials for the extraction of different compounds. In this study, the performance of a novel hydrophobic DES was evaluated for the extraction of phenol from aqueous solutions. Octanoic and dodecanoic fatty acid precursors with a definite molar ratio of 3:1, respectively, were used for the DES having a low melting point of 8.3 °C. The purity and stability of the product were confirmed via characterizing by FTIR, ^1^H and ^13^C NMR methods. The liquid–liquid equilibrium of the water + phenol + DES ternary system at different temperatures of 293.2, 298.2 and 308.2 K was accordingly studied through cloud point titration method and refractive index measurement. Interestingly, the important parameters of the solute distribution coefficient and the separation factor were, respectively, within the high levels of (6.8321–9.7787) and (895.76–2770.17), indicating the amazing capability of the DES. Reasonably, both of these parameters decreased with temperature. The NRTL and UNIQUAC thermodynamic models were employed to reproduce the obtained tie-lines and to determine the interaction parameters at each temperature. The low level root mean square deviations for the mentioned models were, respectively, within (0.0014–0.0027) and (0.0045–0.0063); confirming satisfactorily agreement with the experimental data.

## Introduction

The extensive generation/utilization of phenol in various industries like refineries, petrochemical industries, coal gasification, pulp and paper, paint, pharmaceutical, plastics and resin has leaded to generating huge phenolic wastewaters. Non desired effects of phenol on the environment and human is mainly attributed to its toxicity and hazardous nature. Thus it seems quite beneficial to process wastewaters for separating phenol/phenolic compounds^1^.

Two main strategies for removing phenol from wastewater, reported by the investigators, are (i) degradation via wet oxidation, electrochemical and photocatalytic oxidation and (ii) separation via membrane, extraction and adsorption. Both of these strategies have the benefits and drawbacks. Indeed, recovery and reusability of phenol is not feasible in degradation strategy^2^.

Among various separation techniques such as liquid–liquid extraction, azeotropic distillation and extractive distillation; the latter, due to forming a binary azeotrope between water and phenol, is challenging and thus, liquid–liquid extraction is usually preferred^3^. In this regard, selecting an optimal solvent according to the main criteria of miscibility gap, separation capability, selectivity, environmental friendly and availability is important. In this regard, the undesired properties of most of the volatile conventional solvents such as flammability, toxicity, and regeneration problems have persuaded the researches to design and create novel solvents to overcome these problems^4,5^.

Accordingly, ionic liquids (ILs) and deep eutectic solvents (DESs) as new generations of green solvents have been introduced with desirable properties, e.g. low vapor pressure, non-flammability, biocompatibility and wide liquid phase range^6,7^. Worth mentioning that DESs, in comparison with ionic liquids, have the advantages of easier and cheaper preparation, more biodegradability and less toxicity^8^.

Generally, DESs consist of two or three substances, hydrogen bound donor (HBD) and hydrogen bound acceptor (HBA), bringing about a melting point of less than each substance^9^. In this regard, a typical DES, introduced by Abbot^10^ (2001), was made of choline chloride (HBA) and urea (HBD) with molar ratio of 1:2. Meanwhile, The limitation of utilizing hydrophilic DESs in polar systems encouraged Van Osch et al.^11^ (2015) to propose the first series of hydrophobic DESs for extraction process. Table 1 summarizes the recent investigations on using hydrophobic DESs in the extraction process. Since the involved components significantly influence the DES physico-chemical characteristics, the long hydrocarbon chains e.g. C_8_ and longer fatty acids with the unique properties of non-toxicity and biodegradability^12,13^ are preferred as precursors for preparing DESs^14^. It is while, those made of short hydrocarbon chain fatty acids are usually unstable in water^15^.

**Table 1:** The list of studies on the using of hydrophobic DESs in liquid–liquid extraction.

HBA	HBD	Molar ratio	Reference
Tetraoctylammonium bromide	Decanoic acid	2:1	^11^
Methyltrioctylammonium bromide	Decanoic acid	2:1	
Methyltrioctylammonium chloride	Decanoic acid	2:1	
Tetraoctylammonium chloride	Decanoic acid	2:1	
Tetraheptylammonium chloride	Decanoic acid	2:1	
Tetrabutylammonium chloride	Decanoic acid	2:1	
Menthol	Dodecanoic acid	2:1	^16,17^
Menthol	Palmitic acid	12:1	^18^
n-tetraoctylammonium bromide	Decanoic acid	2:1	^19^
Thymol	Decanoic acid	1:1	
Menthol	Decanoic acid	1:1	
Dodecanoic acid	Menthol	several	^20^
n-nonanoic acid	Menthol	2:1	
Decanoic acid	Menthol	2:1	
Undecanoic acid	Menthol	2:1	
Dodecanoic acid	Decanoic acid	1:2	^14^
Dodecanoic acid	Octanoic acid	1:3	^21^
Dodecanoic acid	Nonanoic acid	1:3	
Thymol	Octanoic acid	1:2	^22^
Menthol	Octanoic acid	1:2	
Thymol	Hexanoic acid	1:1	^23^
Thymol	Heptanoic acid	1:1	
Thymol	Octanoic acid	1:1	
Thymol	Nonanoic acid	1:1	

The desired properties of DESs such as low viscosity and density, flammability and vapor pressure, water immiscibility and stability are remarkable. On the other hand, in order to optimize and simulate the liquid–liquid extraction process, ternary diagrams of the liquid–liquid equilibrium (LLE) must be presented. Here, a novel DES, prepared from octanoic acid (C_8_) and dodecanoic acid (C_12_) precursors was prepared and characterized via FTIR spectroscopy as well as ^1^H and ^13^C NMR analyses. Afterward, the phenol extractability by the proposed DES was scrutinized at different temperatures. Othmer-Tobias, Hand, and Bachman well-known equations were employed to confirm the tie-lines consistency. Using the Aspen Plus simulator, the tie-line data were regressed using the well-known NRTL and UNIQUAC thermodynamic models. For this aim, regarding the composition complexity of the DES; a pioneering novel approach, based on group contribution, was employed for correlating the tie-lines and obtaining the binary interaction parameters.

## Experimental

### Materials

The fatty acid precursors of dodecanoic acid and octanoic acid were purchased from Sigma − Aldrich. Phenol was supplied from Merck and used without additional purification. A water deionizer apparatus (Hastaran, Iran) produced fresh deionized water with conductivity of less than 0.08 μS/cm, utilized for preparing solutions. The chemical names along with other related information are listed in Table 2.

**Table 2:** Specification of the utilized chemicals.

Chemical	CAS No	Supplier	Purity
Water	7732 − 18 − 5	Hastaran	Ultrapure
Phenol	108 − 95 − 2	Merck	> 99%
Octanoic acid	124 − 07 − 2	Sigma − Aldrich	≥ 98%
Dodecanoic acid	143 − 07 − 7	Sigma − Aldrich	98%

### Preparation of DES and solid − liquid phase diagram

**Figure 1:**
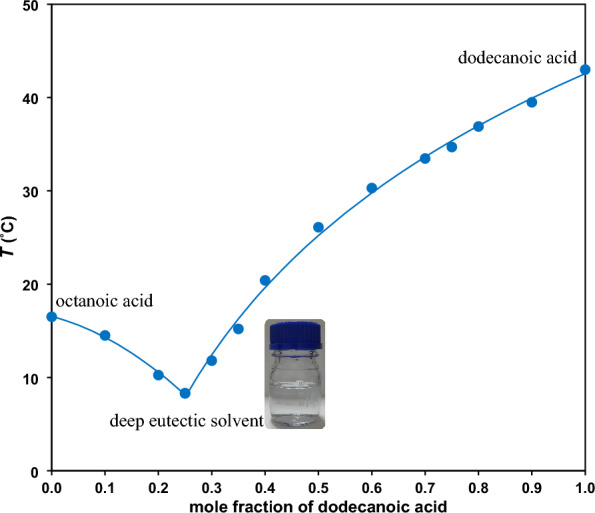
Solid − liquid phase diagram for octanoic and dodecanoic fatty acid precursors.

Different molar ratios of C_8_ and C_12_ carboxylic acids were gradually heated in a jacketed glass vessel up to 70 °C while magnetic stirring until reaching a homogeneous and clear solution of single phase^21,24^. The samples were prepared by mass using an Ohaus balance (Adventurer, Pro AV264, Switzerland, uncertainty 0.0001 g). Consequently, temperature was gradually reduced until the first evidences of solid phase was observed at a certain temperature. The corresponding solid − liquid phase diagram of the DES, illustrated in Fig. 1, shows obtaining a specific DES from octanoic and dodecanoic fatty acids with certain molar ratio of 3:1 (0.25 mol fraction of dodecanoic acid). It can be seen that the melting point of the DES (8.3 °C) is much lower than octanoic acid (16.5 °C) and dodecanoic acid (43.2 °C) bringing about a stable liquid state product for operations under conventional temperatures.

The eutectic product was characterized by FTIR (Perkin Elmer FTIR spectrometer, USA) , ^1^H and ^13^C NMR analysis^16,21^ (Varian − Inova 500 MHz NMR, USA) using dimethyl sulfoxide (DMSO-d6) as solvent. The density and refractive index of the prepared DES and water were measured, respectively, with an oscillating densimeter (Anton Paar DMA4500, Austria) with a relative uncertainty of 0.001, and a refractometer (Abbe AR4 Kruss, Germany) with uncertainty of 0.0007. The obtained values and those reported in literatures are compared in Table 3, indicating a close agreement. Ensuring the stability of the DES in aqueous media, 50% volume ratio in water were stirred for 8 h and was left settling overnight. Therefore, both phases were thoroughly in contact and were separated.

**Table 3:** Refractive index and density for water and the DES at temperature of 298.2 K and under atmospheric pressure of 81.5 kPa^a^.

Component	*n*_*d*_		*ρ* (g/cm^3^)	
	Exp	Lit	Exp	Lit
Water	1.33250	1.33250^25,26^	0.99705	0.99704^27^
				0.99693^28^
DES	1.43050	1.42967^21^	0.90064	0.90100^14^
				0.90110^21^

^a^Average uncertainties are *u*(*T*) = 0.1 K, *u*(*n*_d_) = 0.0007, and *u*_r_(*ρ*) = 0.001.

### Solubility and LLE measurements

The basis of experiments in LLE experiments was the cloud-point titration and refractometry methods. Although various analytical methods have been developed to measure the concentration of components e.g., gas chromatography (GC) and high performance liquid chromatography (HPLC)^29,30^, the most applicable approach to determine the solubility of liquids is cloud point titration, also known as “turbid point titration”. The method has advantages of easily operation, high accuracy and cost effective. An extensive discussion about the conventional methods in LLE analysis has been recently reported by Arce et al.^31^. This approach has been used extensively in recent publications^32,33^. Noteworthy, the visual detection of turbidity and end-point of titration, and the requirement of a calibration curve are the limitation of this analytical method.

For analysis, a set up consisted of a miniature jacketed vial with approximated volume of 5 cm^3^, refractometer, thermostat (Julabo, Germany, uncertainty of 0.01 K) and magnetic stirrer were used. A water stream was circulated by a thermostat driving pump through the jacket and refractometer for maintaining temperature at a certain value. Temperature of the thermostat was calibrated via a reference thermometer (Amadigit, Germany, uncertainty 0.01 K).

Firstly, to determine the calibration curves (relationships between the concentration of each component and the refractive index), the binary mixtures of (water + phenol) or (DES + phenol) with specific amounts were prepared by means of the Ohaus balance. The next step was titrating of the binary mixtures, being stirred at a constant temperature, by precise addition of the remained component (DES or water) via a micro-siring until threshold of a stable cloudy solution. Then, the content was weighed to determine the amount of the third component and refractive index for the cloudy solution was measured immediately. In this way, the calibration curves were attained under atmospheric pressure of 81.5 kPa and different temperatures. In the Supporting materials (Table S1), the calibration curves and related data are presented. It can be seen in Figs. S1 and S2 that the refractive index of cloudy solutions increases with the phenol mass transfer which is attributed to its higher refractive index value.

The equilibrium cells containing different samples with specific and accurate amounts of involved components (water, phenol and DES) were tightly closed. These were agitated in a shaking water bath (N − BIOTEK − 304, South Korea, uncertainty of 0.1 K) at constant temperature and 175 rpm under ambient pressure for 4 h, then the phases were completely separated by resting more than 12 h time.

Collecting the organic phase (top) and aqueous phase (bottom) samples with a syringe, the related refractive indices were determined. It is worth noting that ensuring the accurate experimental data, the analysis of each sample was repeated three times. Using aqueous and organic phase calibration curves and the corresponding refractive indices, the mass fraction of each component with the uncertainty of 0.0002 except *u*(*w*_31_) and *u*(*w*_11_) as 0.0003 was determined. Table 4 presents the tie-line data at different temperatures.

**Table 4:** Experimental tie-line mass fractions (*w*) for water (1) + phenol (2) + DES (3) along with distribution coefficient of water (*D*_1_), of phenol (*D*_2_) and separation factor of phenol (*S*) at different temperatures and under atmospheric pressure 81.5 kPa^a^.

Aqueous phase			Organic phase			*D*_1_	*D*_2_	*S*
*w*_11_	*w*_21_	*w*_31_	*w*_13_	*w*_23_	*w*_33_			
*T* = 293.2 K								
0.9932	0.0042	0.0026	0.0035	0.0406	0.9559	0.0035	9.7787	2770.17
0.9885	0.0090	0.0025	0.0038	0.0765	0.9197	0.0039	8.4983	2205.24
0.9858	0.0118	0.0024	0.0040	0.0961	0.8999	0.0041	8.1368	1989.05
0.9814	0.0164	0.0022	0.0043	0.1262	0.8695	0.0044	7.6931	1745.39
0.9785	0.0194	0.0021	0.0046	0.1462	0.8492	0.0047	7.5361	1618.77
0.9755	0.0225	0.0020	0.0048	0.1684	0.8268	0.0049	7.4844	1528.61
0.9726	0.0255	0.0019	0.0050	0.1897	0.8053	0.0052	7.4392	1437.83
0.9701	0.0281	0.0018	0.0052	0.2084	0.7864	0.0053	7.4164	1392.21
*T* = 298.2 K								
0.9928	0.0043	0.0029	0.0042	0.0395	0.9563	0.0042	9.2045	2198.14
0.9880	0.0093	0.0027	0.0045	0.0741	0.9214	0.0046	8.0045	1744.67
0.9850	0.0124	0.0026	0.0048	0.0943	0.9009	0.0049	7.6091	1547.12
0.9807	0.0168	0.0024	0.0052	0.1248	0.8700	0.0053	7.4154	1407.73
0.9770	0.0208	0.0023	0.0054	0.1527	0.8419	0.0056	7.3573	1321.84
0.9742	0.0236	0.0022	0.0057	0.1729	0.8214	0.0059	7.3181	1246.45
0.9714	0.0265	0.0021	0.0060	0.1931	0.8009	0.0061	7.2863	1188.31
0.9681	0.0299	0.0020	0.0062	0.2162	0.7776	0.0064	7.2304	1125.02
*T* = 308.2 K								
0.9928	0.0043	0.0029	0.0053	0.0365	0.9583	0.0053	8.8729	1676.23
0.9880	0.0093	0.0027	0.0056	0.0670	0.9274	0.0057	7.8353	1379.55
0.9850	0.0124	0.0026	0.0060	0.0964	0.8977	0.0061	7.2643	1197.67
0.9807	0.0168	0.0024	0.0063	0.1217	0.8699	0.0064	7.0973	1104.71
0.9770	0.0208	0.0023	0.0066	0.1486	0.8480	0.0067	6.9739	1037.36
0.9742	0.0236	0.0022	0.0068	0.1674	0.8241	0.0070	6.9513	988.25
0.9714	0.0265	0.0021	0.0071	0.1931	0.7997	0.0074	6.9065	938.47
0.9681	0.0299	0.0020	0.0074	0.2157	0.7797	0.0076	6.8321	895.76

^a^Standard uncertainties are: *u*(*p*) = 0.3 kPa,* u*(*T*) = 0.1 K, *u*(*n*_*d*_) = 0.0007, and u(*w*) = 0.0002 except *u*(*w*_33_) = *u*(*w*_11_) = 0.0003.

The DES capability for selective separation of phenol was evaluated from separation factor (*S*) criterion:

1 $$ S = \frac{{D_{2} }}{{D_{1} }} $$

where *D*_*1*_ is the water distribution coefficient and *D*_*2*_ the phenol distribution coefficients obtained from:

2 $$ D_{1} = \frac{{w_{13} }}{{w_{11} }} $$

3 $$ D_{2} = \frac{{w_{23} }}{{w_{21} }} $$

The greater than unity values of the separation factor and distribution coefficient confirms the capability of the DES for extraction of phenol. A higher *D*_*2*_ is desired because of a higher extracted solute. In other words, less solvent is required to achieve a specific extraction. Also, *S* indicates the separation possibility and the solvent selectivity for the solute extraction^5,34^.

## Results and discussion

### DES characterization

The FTIR spectra of the DES just after preparation and after mixing with water, are presented in Fig. S3. As can be seen, there is no change in DES structure after mixing with water. ^1^H and ^13^C NMR spectra are also given in Supporting materials (Figs. S4 and S5), respectively. Considering no additional peak after mixing with water, it can be concluded that there was no chemical reaction or dissociation of fatty acids, i.e. stable in contact with water. Also, the lack of the precursor peaks in the FTIR and ^1^H NMR of water phase (Fig. S6) confirms again the stability. The summary of the DES analysis data is given in Table 5.

**Table 5:** FTIR, ^1^H NMR and ^13^C NMR spectrums specification for DES and water phases.

Component	Condition	Spectrums	Details
DES	After preparation	FTIR	The peaks at 2856 cm^−1^ and 2926 cm^−1^ represents the stretching of ─CH_2_ and ─CH_3_ vibrations The peak at 1466 cm^−1^ represents the deformation vibration of ─CH2 or ─CH3 groups The peak at of 1712 cm^−1^ represents the stretching vibration of C = O The peak at of 1285 cm^−1^ represents stretching vibration of C─O The peak at of 938 cm^−1^ represents the stretching vibration of ─OH
		^1^H NMR	δ (ppm) = 0.83 (6H, 2CH_3_), 1.22 (24H, 12CH_2_), 1.47 (4H, 2CH_2_), 2.14 (4H, 2CH_2_), 2.49 (DMSO-d_6_), 11.87 (2H, 2OH)
		^13^C NMR	δ (ppm) = 14.16, 22.52, 24.95, 29.04, 31.66, 34.06, 39.57, 174.72
	After mixing with water	FTIR	The peaks at 2856 cm^−1^ and 2927 cm^−1^ represents the stretching of ─CH_2_ and ─CH_3_ vibrations The peak at 1466 cm^−1^ represents the deformation vibration of ─CH2 or ─CH3 groups The peak at of 1712 cm^−1^ represents the stretching vibration of C = O The peak at of 1285 cm^−1^ represents stretching vibration of C─O The peak at of 937 cm^−1^ represents the stretching vibration of ─OH
		^1^H NMR	δ (ppm) = 0.83 (6H, 2CH_3_), 1.22 (24H, 12CH_2_), 1.47 (4H, 2CH_2_), 2.14 (4H, 2CH_2_), 2.49 (DMSO-d_6_), 11.87 (2H, 2OH)
		^13^C NMR	δ (ppm) = 14.15, 22.52, 24.95, 28.94, 31.66, 34.06, 39.89, 174.72
Water	After mixing with DES	FTIR	The peaks at 3435 cm^−1^ represents the stretching vibration of O─H The peaks at 1637 cm^−1^ represents the bending vibration of O─H
		^1^H NMR	δ (ppm) = 2.49 (DMSO-d_6_), 3.53 (2H, 2H_2_O)

### Tie-line results

**Figure 2:**
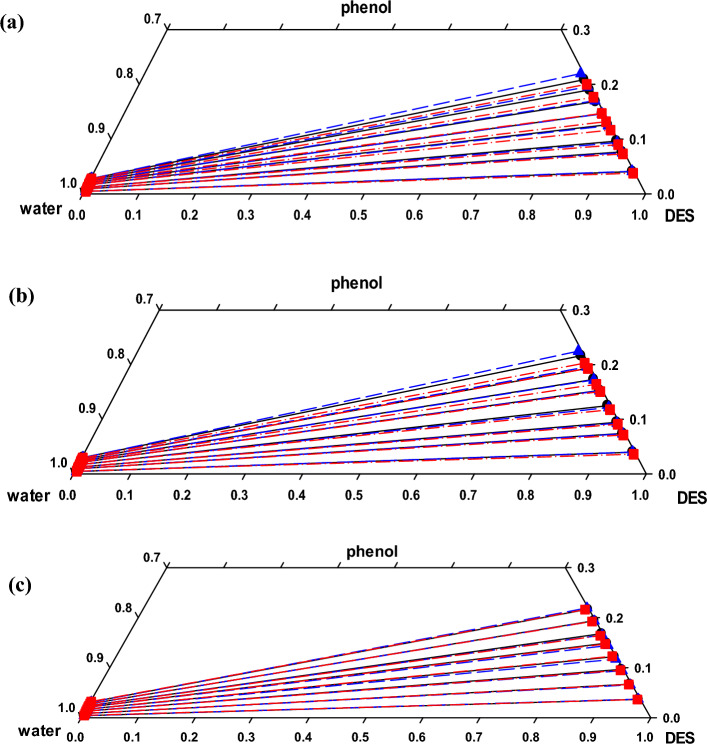
Ternary diagrams of (water + phenol + DES) system at *T* = 293.2 (**a**), 298.2 (**b**) and 308.2 (**c**) K; **●** and solid lines experimental, and dashed lines NRTL predicted tie-lines, and dash-dotted lines UNIQUAC predicted tie-lines.

The tie-line data for the ternary system of water + phenol + DES at different temperatures of 293.2, 298.2 and 308.2 K are listed in Table 4. Corresponding phase diagrams are presented in Fig. 2. In general, mass fractions of the *i*th component of water = 1, phenol = 2 and DES = 3 are presented as *w*_*i*1_ and *w*_*i*_ in the aqueous and organic (DES) phases, respectively. Regarding immiscibility of water and DES, partial miscibility of water and phenol as well as DES and phenol, the investigated system is assigned as a type − 2 LLE system^35^.

As illustrated in Fig. 2, the higher solubility of the solute in the DES phase is consistent with the slope of the obtained tie-lines and can be attributed to the potential hydrogen bonding between phenol and the DES^36,37^. With respect to temperature effect, a gradual diminishing of the binary area is observed with increasing temperature. This can be attributed to the little miscibility tend of water and DES and to some extent, decreasing the phenol hydrogen bound with the DES^38^. It is necessary to mention that the effect of temperature is more evident at higher concentrations of the solute. The same results were previously reported for several chemical systems, for instance, water + phenol + imidazolium ionic liquid^36^, water + phenol + cumene^27^ and water + acetone + HMIMPF_6_ IL^39^.

### Distribution coefficients and selectivity criteria

**Figure 3:**
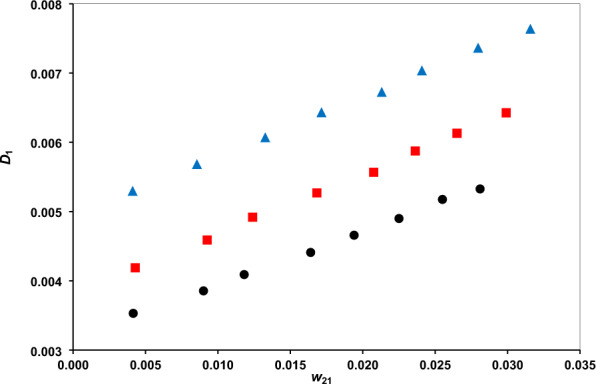
The water distribution coefficients vs. phenol mass fraction at temperatures of 293.2 K (**●**), 298.2 K () and 308.2 K ().

**Figure 4:**
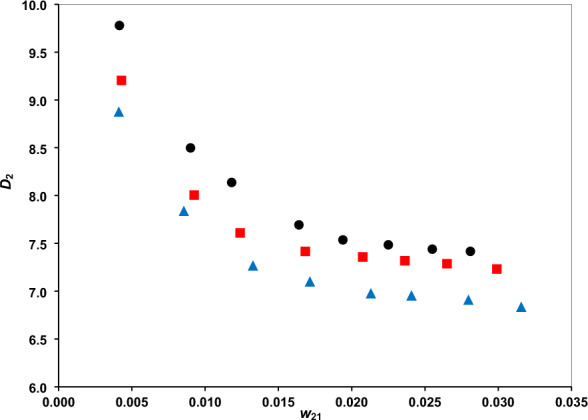
The phenol distribution coefficients of vs. its mass fraction at temperatures of 293.2 K (**●**), 298.2 K () and 308.2 K ().

**Figure 5:**
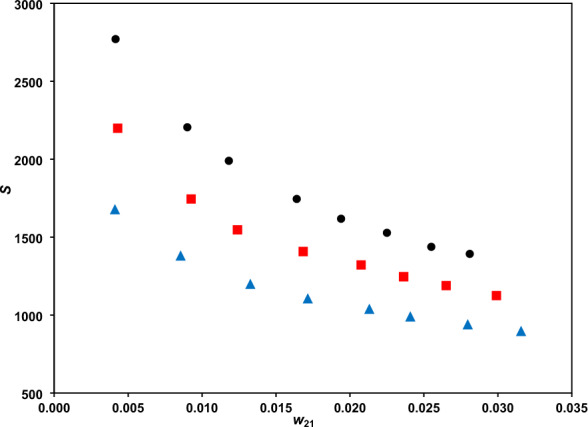
The DES separation factor vs. phenol mass fraction at different temperatures of 293.2 K (**●**), 298.2 K () and 308.2 K ().

The distribution coefficients are presented in Figs. 3 and 4, and the separation factor in Fig. 5. The phenol distribution coefficient lies within the range (6.8321–9.7787) and the separation factor within (895.76–2770.17). These confirm the amazing capability of the DES for phenol extraction. The comparison of separation factor ranges of DES with several other solvents, examined for separation phenol from water, at a typical temperature of 298.2 K, is provided in Table 6. There is significantly higher separation factor for the DES than ILs and some other conventional solvents. Indeed, the higher separation factor of methyl tert-butyl ketone should be regarded with the environmental issues which limits the applications.

**Table 6:** Separation factor for several solvents in phenol extraction from aqueous phase at 298.2 K.

System	Solvent	Separation factor (Ref.)
Water + phenol + cumene	Cumene	93.41–240.58^27^
Water + phenol + imidazolium IL	[Hmim][NTf2]	134.68–948.75^36^
Methyl tert − butyl Ketone + phenol + water	Methyl tert − butyl ketone	1334–3953^40^
Water + phenol + dibutyl ether	Dibutyl ether	50.53–1182.39^41^
Water + phenol + 1-octanol	1-octanol	99.5–495.5^42^
Water + phenol + cyclohexanone	Cyclohexanone	126.3–168.0^42^
Water + phenol + 2-ethyl-1-hexanol	2-ethyl-1-hexanol	131.9–398.1^42^
Mesityl oxide + phenol + water	Mesityl oxide	1007–2599^43^
Choline IL + phenol + water	[choline][NTF2]	27.8–41.3y^44^
Water + phenol + DES	DES	1125.02–2198.14 (present work)

**Figure 6:**
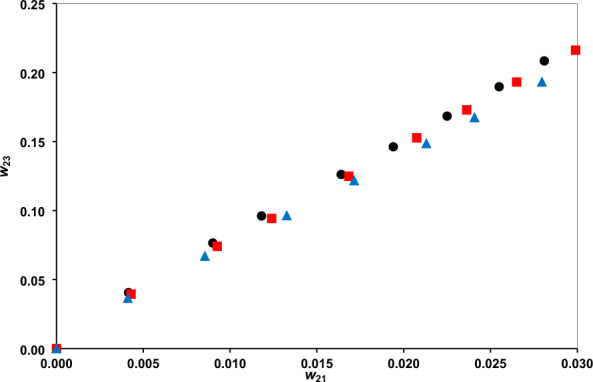
Equilibrium distribution of phenol between the DES and water phases at different temperatures of 293.2 K (**●**), 298.2 K () and 308.2 K ().

The separation factor decreases with increasing phenol mass fraction in aqueous phase (Fig. 5). The reason is relevant to the fact that the water content in the organic DES phase increases with solute concentration. On the other hand, increasing temperature leads to a lower separation factors because of weakening the hydrogen bond between phenol and the DES^38^. Figure 6 presents the phenol equilibrium distribution between the organic (DES) and aqueous (water) phases at different temperatures, showing rather linear variations.

### Consistency tests

The reliability of the experimental data was evaluated based on the Othmer − Tobias, Hand and Bachman well-known correlations as

4 $$ \ln \left( {\frac{{1 - {\text{w}}_{33} }}{{{\text{w}}_{33} }}} \right) = {\text{A}}_{1} {\text{ + B}}_{1} \ln \left( {\frac{{1 - {\text{w}}_{11} }}{{{\text{w}}_{11} }}} \right) $$

5 $$ \ln \left( {\frac{{{\text{w}}_{21} }}{{{\text{w}}_{11} }}} \right) = {\text{A}}_{2} {\text{ + B}}_{2} \ln \left( {\frac{{{\text{w}}_{23} }}{{{\text{w}}_{33} }}} \right) $$

6 $$ w_{33} = A_{3} + B_{3} \left( {\frac{{w_{33} }}{{w_{11} }}} \right) $$

where *A*_*1*_ and *B*_*1*_, *A*_*2*_ and *B*_*2*_, and *A*_*3*_ and *B*_*3*_ are, respectively, intercepts and slopes of the Othmer − Tobias, the Hand and the Bachman correlations^31^. The value of intercepts, slopes and related determination coefficients (*R*^*2*^*)* are tabulated in Table 7. The close to unity determination coefficient verifies the tie-line data consistency.

**Table 7:** The parameters for Othmer − tobias, Hand and Bachman correlations and the corresponding regression coefficients for (water + phenol + DES) system at different temperatures.

*T* (K)	Othmer − Tobias			Hand			Bachman		
	$A_{1}$	$B_{1}$	$R^{2}$	$A_{2}$	$B_{2}$	$R^{2}$	$A_{3}$	$B_{3}$	$R^{2}$
293.2	2.7150	1.1596	0.9995	–2.1192	1.0238	0.9958	–0.1206	1.1181	0.9999
298.2	2.8509	1.2053	0.9997	–2.0861	1.0592	0.9970	–0.1196	1.1162	0.9999
308.2	2.8305	1.2218	0.9992	–2.0416	1.0377	0.9968	–0.1294	1.1259	0.9999

### Correlation models

The consistency of the attained ternary data against the NRTL and UNIQUAC thermodynamic models was checked by utilizing Aspen Plus (V. 8.4) simulator through introducing DES as a pseudo component. Hence, the physico-chemical properties of the DES including normal boiling point, molecular weight and density were introduced to the software. For this aim the method proposed by Mirza et al.^45^ was employed. Relevantly, simultaneous utilization of the modified Lydersen − Joback − Reid (LJR) method along with the mixing rules has been proposed for critical properties estimation as well as normal boiling points. The accuracy of the method has been tested for 39 different DESs^46^. In this regard, group contribution method and the type and frequency of the groups of atoms were considered. The contributions are summated to obtain the final estimate of the boiling point as

7 $$ T_{b} = 198.2 + \sum {n_{i} \Delta T_{bMi} } $$

where $T_{b}$ (K), $n_{i}$ and $\Delta T_{bMi}$(K) are, respectively, the normal boiling point of a DES, the frequency of appearance of the ith group of atoms in the molecule, and group contribution in boiling point for the modified LJR method^45^. Based on Eq. (7) the DES normal boiling point was obtained as 551.0 K.

In the NRTL thermodynamic model, the non-randomness parameter (*α*_ij_) was considered to be 0.2 and 0.3^47^. In the UNIQUAC model, the *r*_*i*_ parameter of the number of segments in each molecule and the *q*_*i*_ parameter of the relative surface area per molecule are listed in Table 8^48^.

**Table 8:** UNIQUAC structral parameters.

Component	*r*	*q*
Water	0.0092	1.4000
Phenol	3.5517	2.6800
DES	27.6928	23.4080

The corresponding interaction parameters of the both models at different temperatures are listed in Table 9. In a previous study, the proper description of the binary interaction parameters are provided^49^. The LLE phase diagram of ternary system based on the obtained data and those of the predicted values by the models are depicted in Fig. 2. Obviously, the models reasonably fit the experimental data.

**Table 9:** Interaction parameters obtained from NRTL and UNIQUAC models for water (1) + phenol (2) + DES (3) system at different temperatures.

*T* (K)	*i − j*	NRTL				UNIQUAC		
		$b_{ij}^{{}}$(K)	$b_{ji}$(K)	$\alpha_{ij}$	RMSD	$a_{ij}^{{}}$(K)	$a_{ji}$(K)	RMSD
293.2	1 − 2	1022.69	465.63	0.2	0.0027	199.22	–2655.13	0.0063
	1 − 3	2299.76	735.93	0.3		175.98	–1146.29	
	2 − 3	–780.80	1129.03	0.3		306.63	–411.06	
298.2	1 − 2	1008.16	416.85	0.3	0.0020	480.09	–1098.45	0.0058
	1 − 3	2339.33	677.67	0.2		178.22	–1106.37	
	2 − 3		–757.61	1057.36	0.2		227.34	–214.64
308.2	1 − 2	1027.34	393.92	0.3	0.0014	527.04	–712.97	0.0045
	1 − 3	2322.88	642.03	0.3		181.39	–1101.86	
	2 − 3	–821.307	1077.02	0.3		180.86	–170.61	

The comprehensive root mean square deviation (RMSD) values were determined from the following equation for each of the investigated models and listed Table 9.

8 $$ RMSD{ = }\sqrt {\frac{{\sum\limits_{{\text{i}}} {\sum\limits_{{\text{m}}} {\sum\limits_{{\text{n}}} {({\text{w}}_{{{\text{imn}}}}^{\exp } - {\text{w}}_{{{\text{imn}}}}^{cal} )^{2} } } } }}{{6{\text{N}}}}} $$

where ${\text{w}}_{{{\text{imn}}}}^{\exp }$ and ${\text{w}}_{{{\text{imn}}}}^{cal}$ represent the experimental and the model predicted mass fractions, respectively. Here, the subscripts of *i* = 1, 2, 3 are for the components and *m* = I, II are for the aqueous and organic phases. Further, the n = 1, 2, …, *N* subscript stand for the number of tie-lines. As shown in Table 9, the RMSD values are very low for the NRTL model within (0.0014–0.0027) and for the UNIQUAC within (0.0045–0.0063). Therefore, the more appropriate model for predicting the tie-line data is the NRTL model

## Conclusions

Utilizing a novel hydrophobic DES, consisted of octanoic and dodecanoic acid precursors, the extraction of phenol from aqueous phase was feasible at different temperatures. The liquid state DES was stable for satisfactory extraction operations. Liquid–liquid equilibrium experiments were performed based on the cloud point titration method and refractive index measurements. The results revealed that low amounts of the DES was required for a specific task and that the tie-lines were with positive slopes since phenol tends to be more in the organic phase. Relevantly, the distribution coefficient and separation factor confirmed the high capacity and selectivity of the DES compared to the conventional organic solvents. Higher separation factors were corresponding to the lower temperature which can be attributed to forming hydrogen bond between phenol and the DES. Meanwhile, the consistency of tie line data was appropriately assessed by the Othmer − Tobias and Bachman equations. Finally, the data were correlated by employing a pioneering approach in group contribution calculations relevant to the thermodynamic models of NRTL and UNIQUAC. The appropriate low RMSD values, particularly for the NRTL model, confirmed good agreement with the experimental data.

To extend investigations, capability of the DES in mass transfer of phenol extraction could be scrutinized. For this aim, the viscosity of the solvent and the molecular diffusivity of phenol in the solvent have to be considered.

### Supplementary Information

Supplementary Information.

## Data Availability

Correspondence and requests for data and materials should be addressed to J.S.
