# Supplementary material for: A green hydrophobic deep eutectic solvent for extraction of phenol from aqueous phase
Source: Sci Rep. 2023 Oct 14;13:17449. doi: 10.1038/s41598-023-44600-x (PMC10576737; doi:10.1038/s41598-023-44600-x)
Supplement: Supplementary file 1 — Supplementary Information. [file 41598_2023_44600_MOESM1_ESM.docx]

**Supporting Materials**

**A green hydrophobic deep eutectic solvent for extraction of phenol from aqueous phase**

Javad Saien
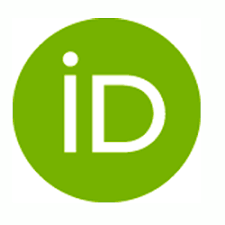

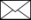
, Mansoureh Bahiraei and Farnaz Jafari

Department of Chemistry and petroleum Science, Bu-Ali Sina University, 65174, Hamedan, Iran

**
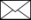
** emails: [saien@basu.ac.ir](mailto:saien@basu.ac.ir), [jsaien@yahoo.com](mailto:jsaien@yahoo.com)

**Supplementary Material**

The Supplementary Material is available at … including table of mass fractions (*w*), and refractive indices (*n*_d_) in cloudy solutions (calibration curves) for the system of water (1) + phenol (2) + DES (3) at different temperatures, Dependency of phenol mass fractions and DES mass fractions in organic and aquoues phase with the refractive index of cloudy solutions, FTIR, ^1^H NMR and ^13^C NMR spectrums of DES after preparation and after mixing with water and FTIR and ^1^H NMR spectrums of water after mixing with DES.

Highlights

Graphical abstract

| aqueous phase | | | |  | organic phase | | | |
| --- | --- | --- | --- | --- | --- | --- | --- | --- |
| w_11_ | w_21_ | *w*_31_ | n_d_ |  | w_13_ | w_23_ | w_33_ | n_d_ |
| *T* = 293.2 K | | | | | | | | |
| 0.9972 | 0.0000 | 0.0028 | 1.33295 |  | 0.0032 | 0.0000 | 0.9968 | 1.43250 |
| 0.9876 | 0.0100 | 0.0024 | 1.33455 |  | 0.0034 | 0.0498 | 0.9468 | 1.43680 |
| 0.9780 | 0.0200 | 0.0020 | 1.33670 |  | 0.0038 | 0.0996 | 0.8966 | 1.44100 |
| 0.9683 | 0.0299 | 0.0018 | 1.33870 |  | 0.0044 | 0.1493 | 0.8463 | 1.44620 |
| 0.9587 | 0.0399 | 0.0014 | 1.34060 |  | 0.0050 | 0.1990 | 0.7960 | 1.45150 |
| 0.9491 | 0.0500 | 0.0010 | 1.34295 |  | 0.0054 | 0.2487 | 0.7460 | 1.45690 |
| 0.9394 | 0.0600 | 0.0006 | 1.34495 |  | 0.0060 | 0.2982 | 0.6958 | 1.46265 |
| 0.9296 | 0.0700 | 0.0004 | 1.34645 |  | 0.0064 | 0.3478 | 0.6459 | 1.46755 |
| 0.9198 | 0.0800 | 0.0002 | 1.34815 |  | 0.0068 | 0.3973 | 0.5959 | 1.47325 |
| *T* = 298.2 K | | | | | | | | |
| 0.9970 | 0.0000 | 0.0030 | 1.33265 |  | 0.0032 | 0.0000 | 0.9968 | 1.43040 |
| 0.9872 | 0.0100 | 0.0028 | 1.33420 |  | 0.0042 | 0.0498 | 0.9460 | 1.43550 |
| 0.9777 | 0.0200 | 0.0024 | 1.33625 |  | 0.0048 | 0.0995 | 0.8957 | 1.44005 |
| 0.9681 | 0.0299 | 0.0020 | 1.33810 |  | 0.0054 | 0.1492 | 0.8454 | 1.44500 |
| 0.9585 | 0.0399 | 0.0016 | 1.34010 |  | 0.0060 | 0.1988 | 0.7952 | 1.45005 |
| 0.9489 | 0.0499 | 0.0012 | 1.34225 |  | 0.0064 | 0.2484 | 0.7452 | 1.45565 |
| 0.9392 | 0.0600 | 0.0008 | 1.34410 |  | 0.0070 | 0.2979 | 0.6951 | 1.46150 |
| 0.9295 | 0.0700 | 0.0005 | 1.34600 |  | 0.0075 | 0.3474 | 0.6451 | 1.46585 |
| 0.9196 | 0.0800 | 0.0004 | 1.34750 |  | 0.0079 | 0.3968 | 0.5952 | 1.47155 |
| *T =* 308.2 K | | | | | | | | |
| 0.9962 | 0.0000 | 0.0038 | 1.33170 |  | 0.0046 | 0.0000 | 0.9954 | 1.42685 |
| 0.9866 | 0.0100 | 0.0034 | 1.33350 |  | 0.0054 | 0.0497 | 0.9449 | 1.43245 |
| 0.9771 | 0.0199 | 0.0030 | 1.33545 |  | 0.0058 | 0.0994 | 0.8948 | 1.43670 |
| 0.9675 | 0.0299 | 0.0026 | 1.33720 |  | 0.0062 | 0.1491 | 0.8448 | 1.44180 |
| 0.9579 | 0.0399 | 0.0022 | 1.33915 |  | 0.0068 | 0.1986 | 0.7946 | 1.44840 |
| 0.9481 | 0.0499 | 0.0020 | 1.34050 |  | 0.0075 | 0.2481 | 0.7443 | 1.45235 |
| 0.9387 | 0.0599 | 0.0014 | 1.34295 |  | 0.0081 | 0.2976 | 0.6943 | 1.45750 |
| 0.9289 | 0.0699 | 0.0012 | 1.34430 |  | 0.0087 | 0.3469 | 0.6443 | 1.46345 |
| 0.9193 | 0.0799 | 0.0008 | 1.34600 |  | 0.0095 | 0.3962 | 0.5943 | 1.46760 |
| *^a^* Standard uncertainties are: *u*(*T*) = 0.1 K, u(*n*_d_) = 0.0007, u(*p*) = 0.3 kPa and u(*w*) = 0.0002 except u(*w_33_*) and u(*w_11_*) = 0.0003. | | | | | | | | |

**Table S1.** Mass fractions (*w*), and refractive indices (*n*_d_) in cloudy solutions (calibration curves) for the system of water (1) + phenol (2) + DES (3) at temperatures of *T* = (293.2, 298.2 and 308.2) K and under the ambient pressure of *p* = 81.5 kPa.*^a^*

**Figure S1.** Dependency of (a) phenol mass fractions and (b) DES mass fractions in DES phase with the refractive index of cloudy solutions at different temperature and *p*=81.5 kPa; (**●**) *T*=293.2 K, (**■**) *T*=298.2 K and (**▲**) *T*=308.2 K**.**

**Figure S2.** Dependency of (a) phenol mass fractions and (b) DES mass fractions in water phase with the refractive index of cloudy solutions at different temperature and *p*=81.5 kPa; (**●**) *T* = 293.2 K, (**■**) *T* = 298.2 K and (**▲**) *T* = 308.2 K**.**

| **(a)**  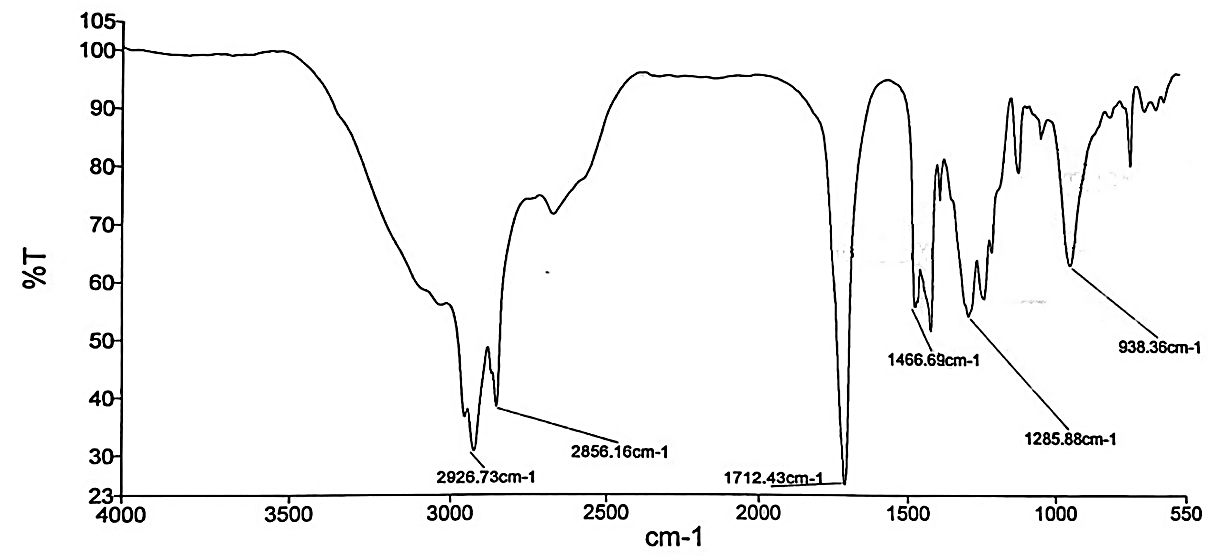 |
| --- |
| **(b)**  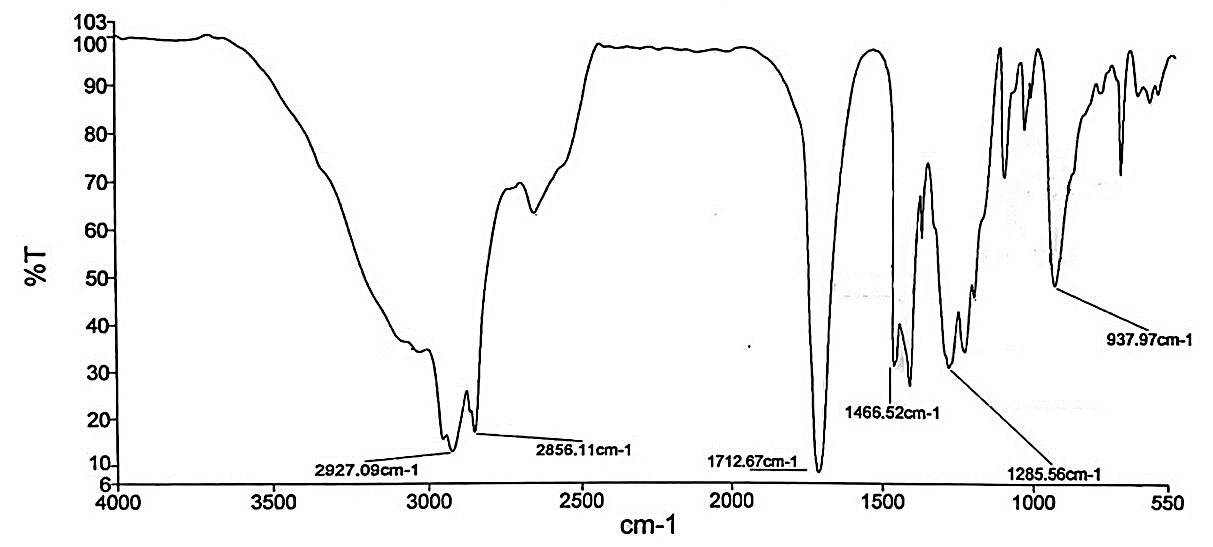 |

**Figure S3.** FTIR spectrums of (a) DES after preparation and (b) DES after mixing with water.

| (a)  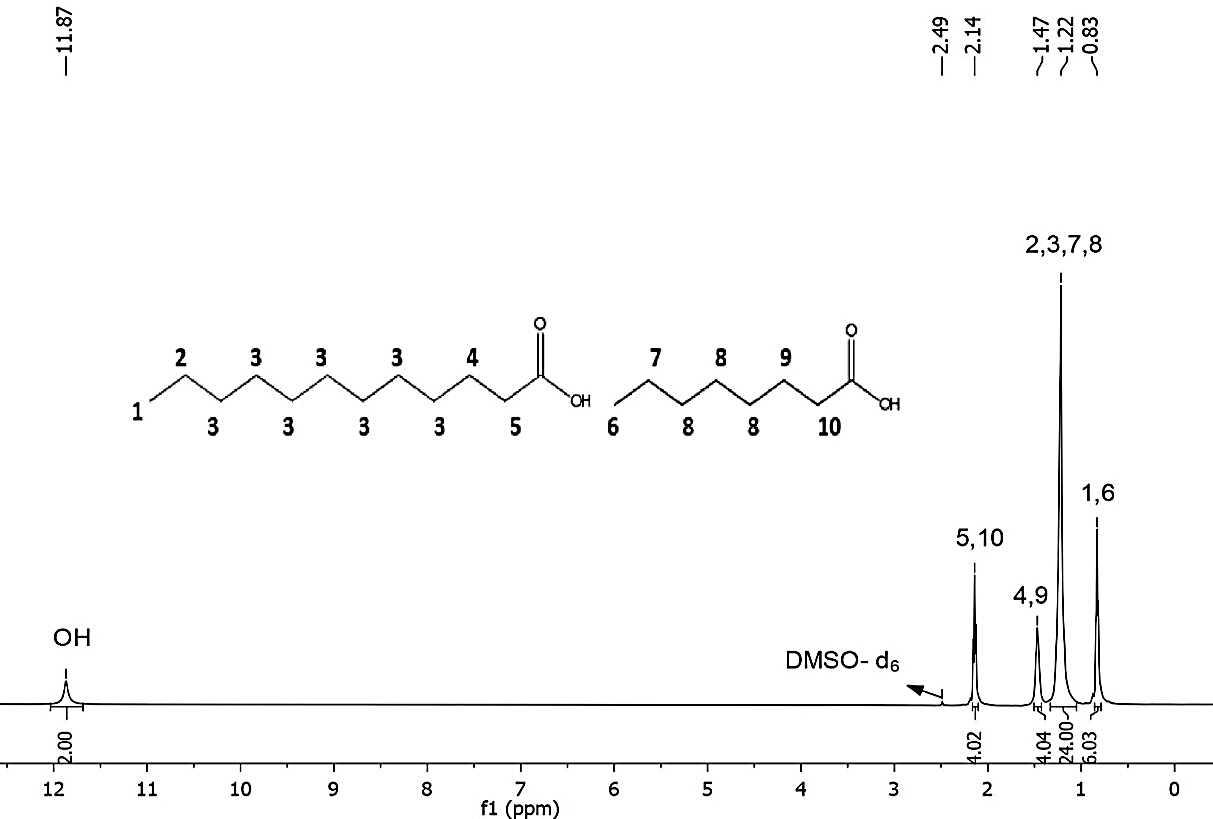 |
| --- |
| (b)  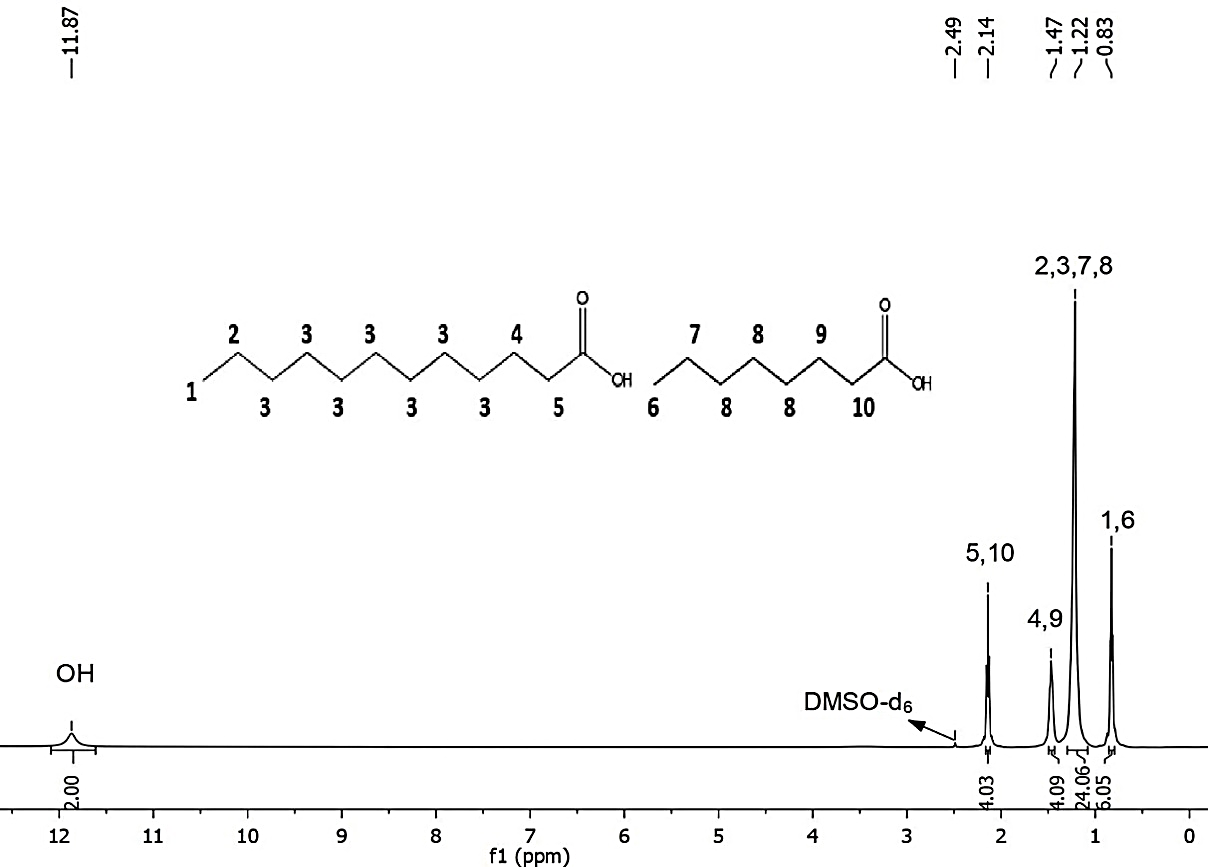 |

**Figure S4.** ^1^H NMR spectrums of (a) DES after preparation and (b) DES after mixing with water.

| (a)  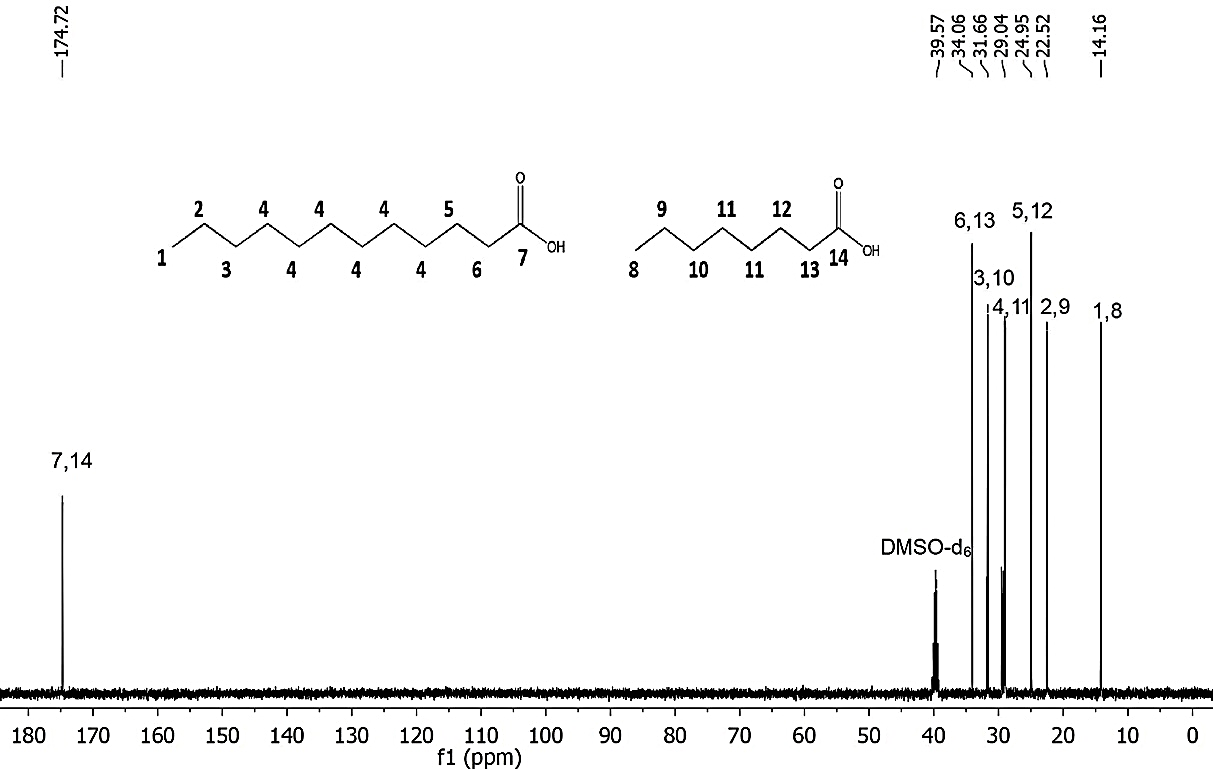 |  |
| --- | --- |
| (b)  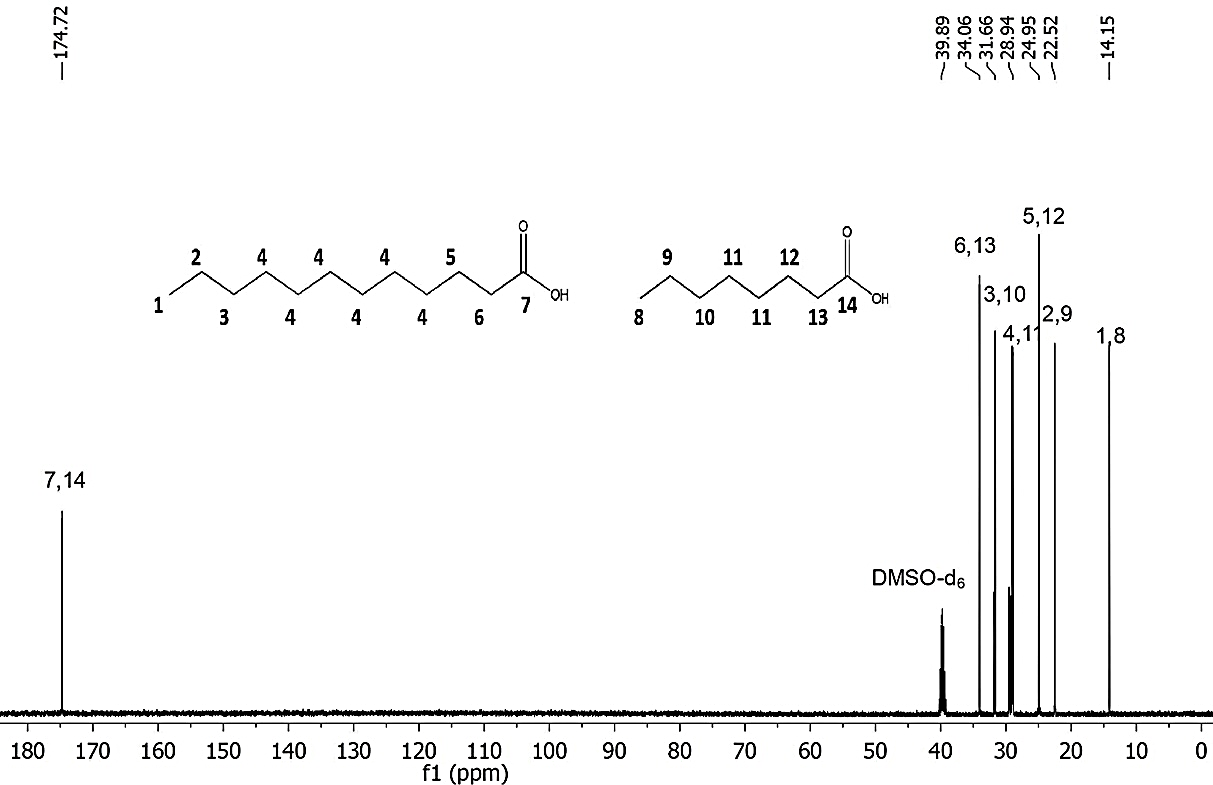 |  |

**Figure S5.** ^13^C NMR spectrum of (a) DES after preparation and (b) DES after mixing with water.

| (a)  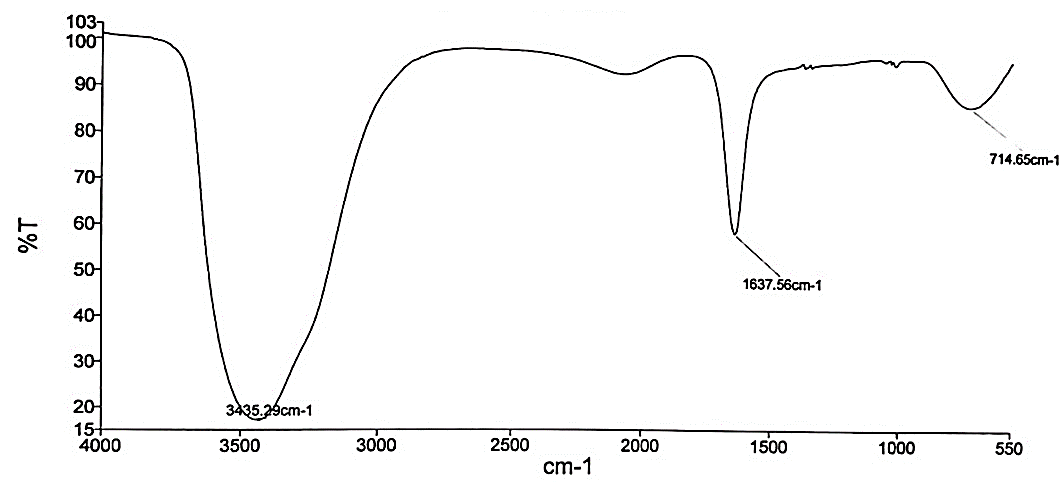 |
| --- |
| (b)  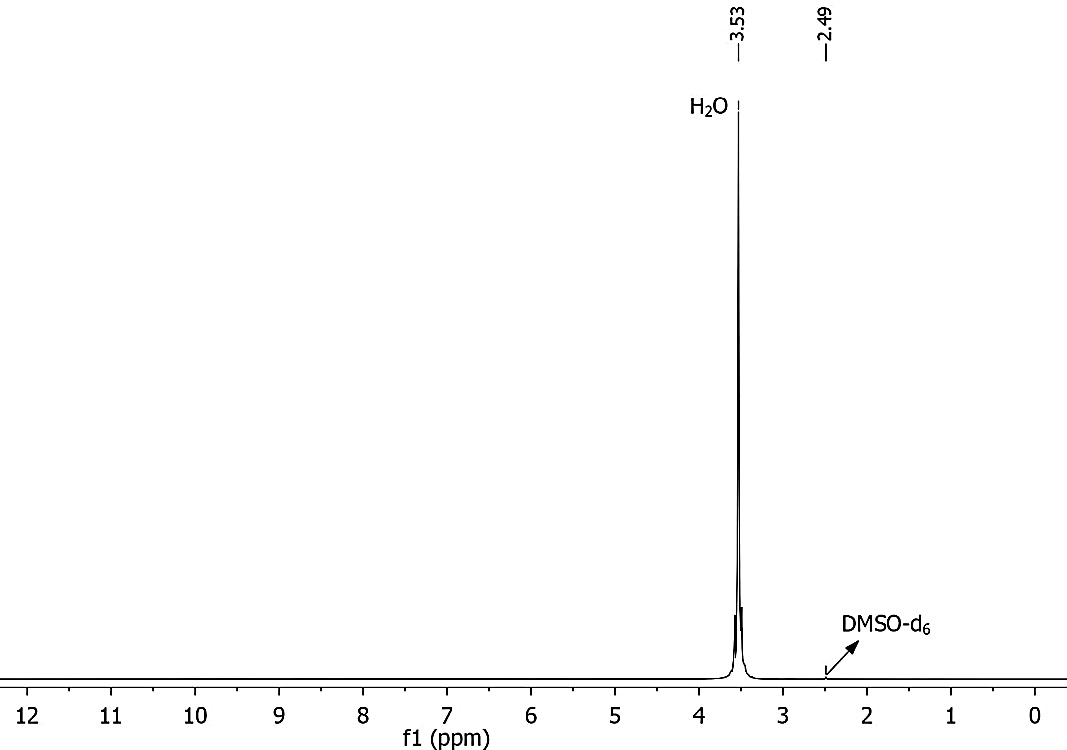 |

**Figure S6.** (a) FTIR and (b) ^1^H NMR Spectrums of water after mixing with DES.

.

**Highlights**

- A novel hydrophobic DES based on carboxylic acids was prepared and characterized.
- Phase behavior of the ternary system of water + phenol + DES was studied at different temperatures.
- High capacity and selectivity were corresponding in phenol separation compared to other solvents.
- The binary interactions of the chemical system were determined based on NRTL and UNIQUIAC models.

**Graphical Abstract**


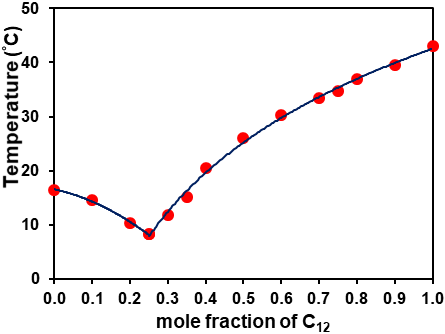

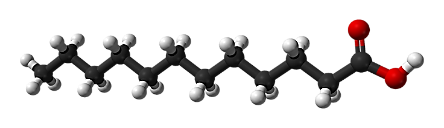

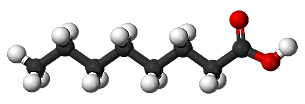

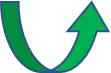

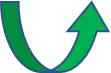

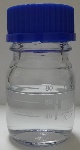


**Eutectic Point**


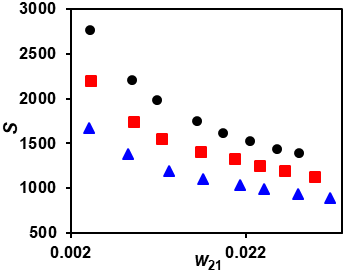


**octanoic acid (C_8_)**

**dodecanoic acid (C_12_)**

**● Experimental Data 298.2 K**

**▲ Predicted Data with NRTL**

**■ Predicted Data with UNIQUAC**

**● 293.2 K**

**■ 298.2**

**▲ 308.2**

1111
